# Performance of the SarQoL quality of life tool in a UK population of older people with probable sarcopenia and implications for use in clinical trials: findings from the SarcNet registry

**DOI:** 10.1186/s12877-022-03077-5

**Published:** 2022-04-27

**Authors:** Miles D. Witham, Philip Heslop, Richard M. Dodds, Andrew P. Clegg, Suzy V. Hope, Claire McDonald, David Smithard, Bryony Storey, Ai Lyn Tan, Anna Thornhill, Avan A. Sayer

**Affiliations:** 1 AGE Research Group, NIHR Newcastle Biomedical Research Centre, Campus for Ageing and Vitality, Newcastle upon Tyne, NE4 5PL UK; 2 Academic Unit for Ageing and Stroke Research, University of Leeds, Bradford Teaching Hospitals NHS Foundation Trust, Bradford, UK; 3 College of Medicine and Health, University of Exeter, and Royal Devon & Exeter NHS Foundation Trust, Exeter, UK; 4 Department of Geriatrics, Gateshead Health NHS Foundation Trust, Gateshead, UK; 5 Queen Elizabeth Hospital, Lewisham and Greenwich NHS Trust and University of Greenwich, London, UK; 6 NIHR Leeds Biomedical Research Centre, Chapel Allerton Hospital, and Leeds Institute of Rheumatic and Musculoskeletal Medicine, University of Leeds, Leeds, UK; 7 Academy of Research and Improvement, Solent NHS Trust, Portsmouth, UK

**Keywords:** Sarcopenia, Quality of life, Validity, Responsiveness, Minimum clinical important difference

## Abstract

**Background:**

The Sarcopenia Quality of Life (SarQoL) questionnaire is a disease-specific sarcopenia quality of life tool. We aimed to independently assess SarQoL with a particular focus on its suitability as a clinical trial outcome measure.

**Methods:**

We analysed data from the UK Sarcopenia Network and Registry. Measures of physical performance and lean mass were collected at baseline. SarQoL and the Strength, Assistance, Rise, Climb - Falls (SARC-F) questionnaire (to assess functional ability) were collected at both baseline and six-month follow-up. Global changes in fitness and quality of life at 6 months were elicited on seven-point Likert scales. Internal consistency was assessed using Cronbach’s alpha. Responsiveness (Cohen’s d and Guyatt coefficients) and minimum clinically important differences were calculated for participants reporting slight improvement or worsening in their global scores. Concurrent validity was assessed by correlating baseline SarQoL scores with measures of physical performance and functional ability.

**Results:**

We analysed data from 147 participants, 125 of whom underwent follow up assessment; mean age 78 years; 72 (49%) were women. Internal consistency was good; Cronbach’s alpha was 0.944 at baseline and 0.732 at telephone follow-up. Correlation between baseline and follow-up SarQoL was weak (*r* = 0.27; *p* = 0.03). The minimum clinically important improvement ranged from 5 to 21 points giving trial sample size estimates of 25–100 participants. SarQoL scores were moderately correlated with handgrip (*r* = 0.37; *p* < 0.001), SARC-F (*r* = − 0.45; *p* < 0.001), short physical performance battery (*r* = 0.48; *p* < 0.001) and 4-m walk speed (*r* = 0.48; *p* < 0.001).

**Conclusions:**

SarQoL has acceptable performance in older UK participants with probable sarcopenia and is sufficiently responsive for use in clinical trials for sarcopenia.

## Introduction

Sarcopenia, the age-related loss of muscle mass and function, is common and clinically important [1]. It is associated with an increased risk of falls, future disability and dependency, hospital admission and earlier death [2–4]. Finding new ways to prevent or treat sarcopenia is therefore an important area of research. Conducting clinical trials for sarcopenia is however not straightforward. Measuring muscle mass and muscle function are important efficacy outcomes in sarcopenia trials, but health related quality of life is also a key consideration [5]. Whilst there are many generic tools for measuring health related quality of life, until recently tools designed specifically for measuring quality of life in people with sarcopenia were lacking.

The SarQoL questionnaire was introduced in 2015 [6] to fill this gap. Originally derived in French and tested with older people from a Belgian outpatient clinic, it has been translated into multiple languages including English [7]. The questionnaire consists of 55 items organised into 22 questions across seven domains: Physical and mental health, Locomotion, Body composition, Functionality, Activities of daily living, Leisure activities and Fears.

Although some validation work has been performed [8–10], there is a need to test the internal consistency, responsiveness (how much the measure changes when a real change in health occurs) and concurrent validity (how the measure compares to measures that would be expected to be related to the measure under test) in a UK population of older people with sarcopenia. In particular, there is a need to derive the minimum clinically important difference for SarQoL using currently recommended anchor-based methods [11] to enable sample size calculations for clinical trials, but also to enable interpretation of effect sizes derived from intervention studies.

The aim of this analysis was therefore to test the internal consistency, responsiveness and concurrent validity of the SarQoL tool in a cohort of older people with sarcopenia in the UK.

## Methods

### Study population

We analysed data from the UK Sarcopenia Network and Registry (SarcNet) pilot study; the study design and baseline data have been reported previously [12]. SarcNet was designed as an observational study with a baseline visit and a six-month follow up. The target population was people aged 65 and over with self-reported impairment in physical function. To ensure that as many potentially eligible participants were included in SarcNet, we used a SARC-F score [13] of 3 or more out of 10 at telephone pre-screening (in line with our previous LACE randomised controlled trial and with recent data on the ability of SARC-F to detect patients with probable sarcopenia in similar populations) [13–15] rather than the more commonly used cutoff of 4. Participants were recruited from primary care organisations (General Practices) in the UK and assessed at six hospital-based recruitment sites. Exclusion criteria were life expectancy of less than 6 months in the judgement of the local investigator, participation in an interventional study within the last 30 days. Other exclusion criteria were: presence of a permanent pacemaker with an atrial sensing lead or presence of an implantable cardioverter-defibrillator, peripheral oedema present above knee level or fever at the baseline visit (all contraindications to bioimpedance testing). Previous analysis of baseline data [12] showed that 94% of those in SarcNet fulfilled the 2019 European Working Group on Sarcopenia in Older People (EWGSOP) criteria [16] for probable sarcopenia.

### Measures collected

The SarQoL questionnaire (in its English translation) [7] was administered by the research nurse as part of the SarcNet study at baseline and at six-month follow-up to assess quality of life. Baseline visits were conducted face-to-face (in a research clinic or in the participants own home). Due to limitations on face-to-face research activity imposed in the UK as part of the pandemic response to COVID-19, all but eight follow-up visits were conducted by telephone by the research nurses. The SARC-F score was collected by telephone at pre-screening and at the six-month follow-up encounter (whether face to face or by telephone) by the research nurses.

At the baseline visit, maximum hand grip strength was measured using a Jamar hydraulic dynamometer (Lafayette Instrument Company, USA) [17]. Three measurements were taken on each hand and the maximum value was used for analysis. Appendicular lean muscle mass was measured using the Akern 101 bioimpedance system (Akern SRL, Pontassieve, Italy). Resistance and reactance were recorded and the Sergi equation [18] was used to derive appendicular skeletal muscle mass index (appendicular skeletal muscle mass divided by height squared). The Short Physical Performance Battery (SPPB) [19] was conducted, comprising side-by-side, semi-tandem and tandem balance tests, gait speed over 4-m walk distance, and five times sit to stand time from a chair without using arms to assist. At the six-month follow-up visit, all participants were asked two questions to assess global change in a) fitness, and b) quality of life. Change in fitness was assessed by the response on a 7-point Likert scale (much worse to much better) to the statement “Since the first visit, my overall fitness is…”. Change in quality of life was assessed by the response on a similar 7-point Likert scale to the statement “Since the first visit, my overall quality of life is…”. Due to only small numbers of participants recording the most extreme changes on the Likert scale, responses for ‘much better’ and ‘much worse’ were amalgamated with ‘better’ and ‘worse’ respectively. Few participants reported minimal global improvement, thus an additional category of ‘any improvement’ (including those reporting slightly better, better or much better) was also derived.

### Statistical analysis

All statistical analyses were conducted using SPSS v26 (IBM, New York, USA). A two-sided *p* value of < 0.05 was taken as significant for all analyses. Descriptive statistics were generated for the full baseline group, those that dropped out before 6 months, and those that underwent follow up by telephone and face to face at 6 months; means and standard deviations were reported for normally-distributed continuous variables, medians and interquartile ranges were reported for continuous variables that were not normally distributed on visual inspection. Baseline characteristics of those undergoing telephone follow-up were compared with those undergoing face-to-face follow-up and with those who did not undergo assessment at 6 months; Student’s t-test and Mann-Whitney U tests were used for normally and non-normally distributed continuous variables, and Pearson’s chi-squared test was used to compare categorical variables. The internal consistency (a measure of whether individual questionnaire items are related to each other) of SarcQol was analysed using Cronbach’s alpha. The baseline, follow-up and telephone follow-up populations were analysed separately, and internal consistency within each of the SarQoL domains was also assessed. Correlations between each subdomain and both the total baseline SarQoL score and the score without the index subdomain were calculated using Pearson’s correlation coefficient to test whether a single subdomain could substitute for the total score.

Correlation between baseline and six-month follow-up values was assessed using Pearson’s correlation coefficient. This value is a key factor in calculating sample sizes for trial analyses that adjust for baseline values and is more informative for planning trial sample sizes than the intraclass correlation coefficient commonly calculated as part of psychometric assessment of measurement tools. A high correlation between baseline and follow-up values (i.e. a more stable measure) enables greater precision in detecting changes between groups, and the sample size can be reduced according to the formula (1 - r^2^) for a single time-point follow-up trial [20]. Responsiveness to change was calculated in two ways. Cohen’s d (equivalent to effect size) was calculated as the mean SarQoL difference between baseline and follow up) / pooled SD of baseline and follow up SarQoL [21]. Cohen’s d was calculated separately for groups reporting a slight improvement, a slight worsening, or any improvement. Guyatt’s responsiveness coefficient was calculated for the same groups using the mean change in SarQoL score for each group / SD of the change in SarQoL score in the group showing ‘no change’ on the Likert scale [22]. For comparison, responsiveness was also calculated for the SARC-F score in the same way.

Concurrent validity was assessed by calculating the correlation between baseline SarQol scores (total and individual domains), measures of physical performance (maximal grip strength, SPPB score and 4 m walk speed), and function in daily life measured by the SARC-F score. Pearson’s correlation coefficients were calculated as all data were normally distributed. Finally, a range of sample size calculations were performed to show the number of participants that would need to be recruited to detect different Minimum Clinically Important Difference (MCID) values for SarQoL under different assumptions.

## Results

**Fig. 1:**
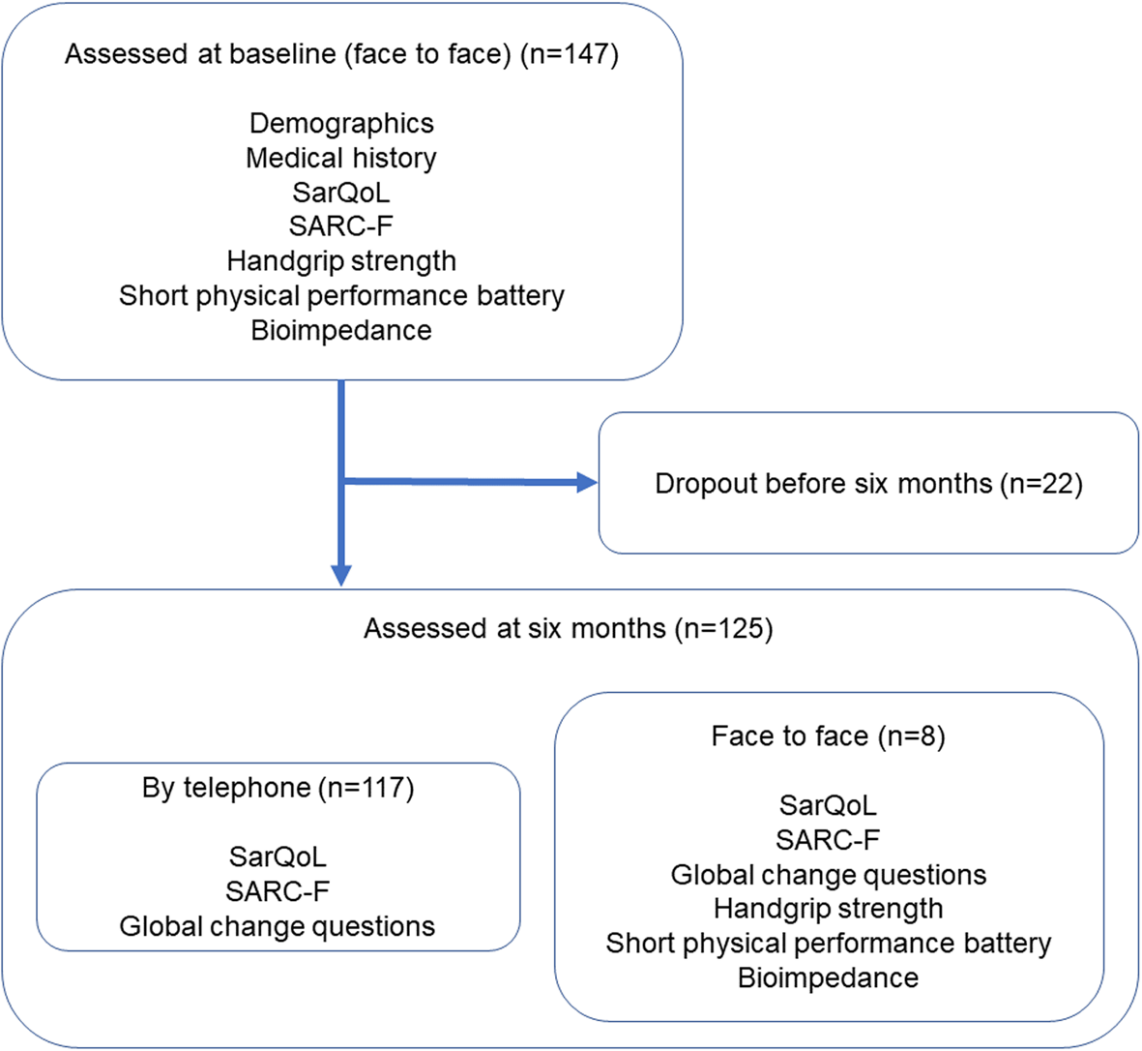
Flow of participants through the study

Data were available for 147 participants at baseline, and 125 participants underwent six-month follow up. Eight follow-up visits were conducted face-to-face; the others were conducted by telephone. The flow of participants through the study is shown in Fig. 1. The mean time between baseline and follow up was 6.5 (SD 1.1) months. Details of the baseline characteristics of the whole group, those undergoing six-month follow-up by telephone or face-to-face, and those who dropped out before six-month follow-up are given in Table 1.

**Table 1:** Baseline characteristics of study population

	All included participants (*n* = 147)	Participants by 6 month follow up status		
		Telephone (*n* = 117)	Face-to-face (*n* = 8)	Dropped out (*n* = 22)
Mean age (years) (SD)	77.6 (7.3)	77.0 (7.2)	78.9 (6.0)	79.8 (7.8)
Female sex (%)	72 (49)	56 (48)	3 (38)	13 (59)
Mean SPPB (SD)	5.5 (2.3)	5.8 (2.4)	6.4 (1.8)	5.6 (2.5)
Max handgrip strength (kg) (SD)				
Men	27.2 (9.4) (*n* = 70)	26.8 (9.2) (*n* = 61)	32.8 (12.6) (*n* = 5)	26.6 (9.2) (*n* = 9)
Women	15.7 (6.1) (*n* = 69)	15.5 (6.4) (*n* = 56)	16.7 (3.1) (*n* = 3)	16.5 (5.2) (*n* = 13)
Mean walk speed (m/s) (SD)	0.61 (0.24) (*n* = 142)	0.61 (0.24) (*n* = 113)	0.64 (0.21) (n = 8)	0.62 (0.23) (*n* = 21)
Median chair stand time (s) [IQR]	24.0 [32.0–17.4] (*n* = 106)	23.0 [14.5] (*n* = 84)	21.8 [26.0] (*n* = 6)	28.0 [7.6] (*n* = 16)
Mean SARC-F score (SD)	5.28 (1.82)	5.28 (1.85)	4.38 (1.19)	5.59 (1.82)
Mean skeletal muscle mass index (kg/m^2^) (SD)				
Men	7.84 (1.21) (*n* = 70)	7.82 (1.11) (*n* = 57)	7.75 (0.92) (*n* = 5)	8.03 (2.01) (*n* = 8)
Women	6.65 (1.13) (*n* = 69)	6.76 (1.23) (*n* = 54)	6.12 (0.30) (*n* = 3)	6.30 (0.61) (*n* = 12)
SarQoL score (SD) (best = 100)	50.9 (11.9)	51.1 (12.6)	52.6 (7.6)	49.5 (9.3)
Physical and mental health	55.5 (15.6)	55.4 (15.9)	57.9 (7.8)	55.3 (16.3)
Locomotion	48.8 (18.1)	49.1 (18.7)	43.8 (12.8)	48.8 (17.2)
Body composition	58.4 (17.2)	58.0 (17.7)	59.9 (12.4)	59.8 (16.8)
Functionality	52.6 (12.8)	52.7 (13.5)	55.1 (7.7)	51.4 (10.6)
Activities of daily living	46.1 (14.9)	46.2 (15.4)	52.3 (10.9)	43.3 (13.1)
Leisure activities	36.1 (17.3)	36.5 (18.2)	31.2 (5.9)	36.3 (15.1)
Fears	67.5 (19.1)	68.9 (19.8)	68.8 (22.2)	59.7 (11.5)*

*p < 0.05 compared to telephone follow-up group

Subdomain scores all normalised to 0–100 scale (best = 100)

### Internal consistency and subdomain correlations

Table 2 shows the results of internal consistency testing using Cronbach’s alpha. The full SarQoL score showed acceptable levels of consistency at baseline (alpha = 0.944) but showed less consistency at the follow up visit (alpha = 0.732). No differences in consistency were seen when confining the follow up analysis only to those undergoing telephone assessment at the six-month visit. Although consistency within most subdomains was good, consistency within the body composition and leisure activities domains was poor. Table 3 shows correlations between each subdomain and the total baseline score; the functionality and activities of daily living domains had the highest correlation with the total score.

**Table 2:** Internal consistency of SarQoL and subdomains (by Cronbach’s alpha)

	Baseline data (*n* = 144) (95% CI)	Follow up data (*n* = 125) (95% CI)	Telephone follow up data (*n* = 117) (95% CI)
SarQoL total	0.94 (0.93, 0.96)	0.71 [0.63,0.78)	0.73 [0.66, 0.80)
Physical and mental health	0.76 (0.70, 0.82)	0.83 [0.79, 0.87)	0.83 [0.78, 0.87)
Locomotion	0.89 (0.86, 0.92)	0.30 [0.10, 0.47)	0.28 [0.64, 0.46)
Body composition	0.17 (−0.07, 0.37)	0.09 [− 0.21, 0.32)	0.09 [− 0.22, 0.33)
Functionality	0.87 (0.83, 0.90)	0.86 [0.83, 0.90)	0.86 [0.82, 0.89)
Activities of daily living	0.88 (0.85, 0.91)	0.88 [0.85, 0.91)	0.88 [085, 0.91)
Leisure activities	0.39 (0.15, 0.56)	0.45 [0.22, 0.61)	0.42 [0.17, 0.60)
Fears	n/a^a^	n/a^a^	n/a^a^

^a^unable to calculate as only one question in this domain

**Table 3:** Pearson’s correlation between each subdomain of SarQoL and total score at baseline

Subdomain	Correlation with total SarQoL score		Correlation with mean of all subdomains excluding the subdomain under test	
	*r*	*p*	*r*	*p*
Physical and mental health	0.80	< 0.001	0.72	< 0.001
Locomotion	0.84	< 0.001	0.66	< 0.001
Body composition	0.59	< 0.001	0.52	< 0.001
Functionality	0.88	< 0.001	0.74	< 0.001
Activities of daily living	0.87	< 0.001	0.66	< 0.001
Leisure activities	0.33	< 0.001	0.28	< 0.001
Fears	0.32	< 0.001	0.24	< 0.001

### Change in SarQoL over time

**Fig. 2:**
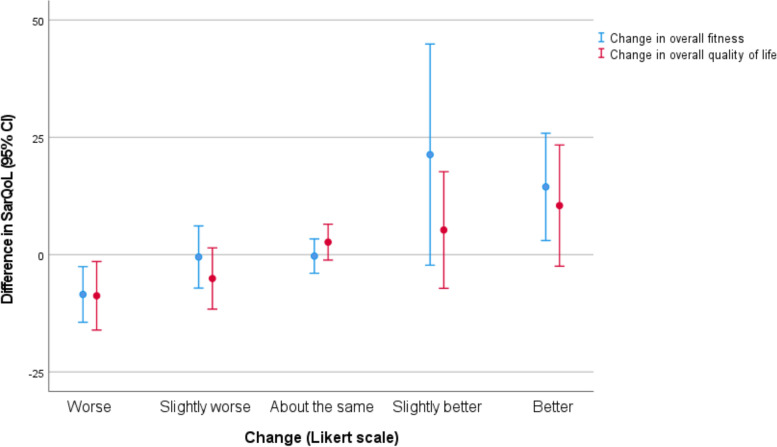
Relationship between global change and mean change in SarQol score between baseline and follow-up

Follow up SarQoL scores at 6 months were weakly correlated with scores at baseline (*r* = 0.27, *p* = 0.03). In contrast, follow-up SARC-F scores were much more closely correlated with baseline SARC-F scores (*r* = 0.63, *p* < 0.001). Figure 2 shows the changes in the SarQoL score between baseline and follow-up for each category of global change in fitness or quality of life; details of the changes for each subdomain and for the SARC-F score are shown in Table 4. More participants reported worsening of global fitness or quality of life than reported improvement; no change in global fitness or quality of life were the most commonly selected categories for global change. Point estimates for SarQoL and for SARC-F in those reporting ‘no change’ were close to zero, suggesting no systematic bias in how scores changed between face-to-face assessment at baseline and telephone assessment at follow-up. Of the subdomains, functionality mapped most closely to the total score, both in terms of a stable functionality score in those reporting no global change, and also in the degree of improvement or deterioration in scores.

**Table 4:** Mean Change in SarQol and other measures between baseline and follow-up

A. By global change in overall fitness						
	Worse (*n* = 30)	Slightly worse (*n* = 25)	About the same (*n* = 46)	Slightly better (*n* = 5)	Better (*n* = 11)	Any improvement (*n* = 16)
Overall SarQoL	−8.5 (15.9)	−0.5 (16.0)	−0.3 (12.3)	21.3 (19.0)	14.4 (17.0)	16.6 (17.3)
Physical and mental health	−6.2 (20.4)	2.4 (19.0)	5.8 (17.5)	32.7 (13.1)	17.4 (18.4)	22.1 (18.0)
Locomotion	−7.8 (23.7)	−0.5 (19.2)	−4.3 (23.1)	25.0 (31.7)	16.4 (23.5)	19.1 (25.5)
Body composition	−3.9 (22.5)	4.1 (22.1)	12.4 (25.6)	22.5 (12.0)	14.0 (20.1)	16.7 (18.0)
Functionality	−7.4 (15.5)	−0.7 (17.1)	− 0.9 (11.4)	15.5 (25.5)	14.7 (14.9)	14.9 (17.9)
Activities of daily living	−11.9 (17.2)	−0.9 (19.4)	−4.5 (14.9)	20.5 (17.7)	12.0 (22.3)	14.7 (20.8)
Leisure activities	−11.6 (29.3)	0.7 (17.0)	6.9 (21.8)	20.0 (13.9)	12.1 (19.8)	14.5 (18.1)
Fears	−6.7 (22.9)	3.5 (29.9)	4.9 (32.2)	7.5 (30.1)	6.8 (32.8)	7.0 (30.9)
SARC-F	0.83 (1.49)	0.28 (1.99)	−0.07 (1.67)	−1.00 (2.12)	−1.64 (1.69)	−1.44 (1.79)
B. By global change in overall quality of life						
	Worse (*n* = 26)	Slightly worse (*n* = 15)	About the same (*n* = 63)	Slightly better (*n* = 3)	Better (*n* = 10)	Any improvement (*n* = 13)
Overall SarQoL	−7.6 (17.5)	−5.1 (11.8)	2.7 (15.2)	5.3 (5.0)	10.5 (18.1)	9.3 (15.9)
Physical and mental health	−6.0 (22.1)	−3.0 (19.2)	7.6 (17.7)	18.5 (22.2)	16.3 (21.1)	16.8 (20.4)
Locomotion	−8.4 (26.5)	−11.0 (15.0)	−0.3 (24.0)	5.6 (12.7)	12.9 (25.6)	11.2 (23.0)
Body composition	−3.0 (25.8)	−1.9 (18.5)	11.5 (24.2)	16.7 (7.2)	15.8 (19.8)	16.0 (17.4)
Functionality	−6.4 (17.0)	−5.0 (15.2)	1.5 (15.0)	−1.1 (14.2)	11.1 (16.3)	8.3 (16.2)
Activities of daily living	−13.2 (18.5)	−3.8 (18.8)	0.4 (18.0)	−1.0 (11.5)	4.9 (20.4)	3.6 (18.4)
Leisure activities	−10.9 (30.8)	0.0 (15.4)	5.0 (21.2)	27.7 (9.6)	10.0 (22.4)	14.1 (21.3)
Fears	−9.6 (19.8)	−1.7 (29.8)	7.7 (31.1)	0.0 (25.0)	1.3 (36.1)	1.0 (32.9)
SARC-F	0.60 (1.65)	0.33 (2.22)	−0.08 (1.60)	2.33 (1.53)	−1.70 (1.83)	−0.77 (2.45)

Values are mean and standard deviation

### Responsiveness

Table 5 shows measures of responsiveness calculated separately for ‘slight improvement’ and ‘slight worsening’ in global fitness and global quality of life. Results for all categories of improvement combined are also presented, as numbers in each individual improvement category were small. The SarQoL tended to be more responsive to change than the SARC-F, and both tools were more responsive to improvement than to worsening.

**Table 5:** Responsiveness measures for SarQoL and other measures

	Cohens d for slightly worse	Cohens d for slightly better	Cohens d for any improvement	Guyatt for slightly worse	Guyatt for slightly better	Guyatt for any improvement
A. By global change in overall **fitness**						
SarQoL	0.03	2.29	1.11	0.04	1.73	1.35
SARC-F	0.04	0.48	0.29	0.17	0.60	0.86
B. By global change in overall **quality of life**						
SarQoL	0.36	0.98	0.75	0.34	0.35	0.61
SARC-F	0.06	1.38	0.19	0.21	1.46^a^	0.48

^a^paradoxical worsening

### MCIDs and sample size estimates

Table 6 depicts the anchor-based minimum clinically important differences for the SarQoL and SARC-F, and reports trial sample sizes that would be needed to detect these differences with 80 and 90% power, with and without adjustment for the correlation between baseline and follow-up, given an alpha of 0.05.

**Table 6:** Sample sizes required to detect minimum clinically important differences

		MCID	SD	Sample size			
				80% power, unadjusted	80% power, adjusted	90% power, unadjusted	90% power, adjusted
SarQoL– change in fitness	Slight improvement	21	19	26	24	36	34
	Any improvement	17	17	32	30	44	42
	Slight worsening	1	16	8038	7476	10,760	10,008
SarQoL – change in QoL	Slight improvement	5	5	32	30	44	42
	Any improvement	9	16	100	94	134	126
	Slight worsening	5	12	182	170	244	228
SARC-F – change in fitness	Slight improvement	1.0	2.1	140	86	186	114
	Any improvement	1.4	1.8	52	32	70	44
	Slight worsening	0.3	2.0	1396	852	1868	1140
SARC-F – change in QoL	Slight improvement	2.3	1.5	14	10	18	12
	Any improvement	0.8	2.5	308	188	412	252
	Slight worsening	0.3	2.2	1690	1032	2262	1380

*MCID* Minimum clinically important difference. Sample size is total sample size for a two arm trial. Calculations assume 1:1 randomisation, alpha 0.05. Adjusted: Sample size multiplied by (1 – r^2^) where *r* = 0.27 for correlation between baseline and follow-up SarQoL scores, and *r* = 0.63 for correlation between baseline and follow-up SARC-F scores. Where calculations deliver odd numbers, sample size is rounded up to nearest even number

### Concurrent validity

Table 7 shows correlations between the total SarQoL score, measures of physical performance and the SARC-F score. The SarQoL score showed moderate correlations with each of the other measures as expected.

**Table 7:** Correlation between baseline SarQoL subdomains and baseline measures of physical function

	SARC-F		Max grip		SPPB		Walk speed	
	*r*	*p*	*r*	*p*	*r*	*p*	*r*	*p*
SarQoL total	−0.45	< 0.001	0.37	< 0.001	0.48	< 0.001	0.48	< 0.001
Physical and mental health	−0.35	< 0.001	0.29	0.001	0.33	< 0.001	0.26	0.004
Locomotion	−0.35	< 0.001	0.21	0.19	0.44	< 0.001	0.51	< 0.001
Body composition	−0.27	0.002	0.18	0.05	0.25	0.006	0.19	0.04
Functionality	−0.44	< 0.001	0.34	< 0.001	0.45	< 0.001	0.42	< 0.001
Activities of daily living	−0.43	< 0.001	0.42	< 0.001	0.46	< 0.001	0.48	< 0.001
Leisure activities	−0.09	0.34	0.22	0.02	0.29	0.001	0.26	0.004
Fears	−0.26	< 0.001	0.20	0.02	0.18	0.04	0.11	0.22

## Discussion

Our analysis is the first to validate the SarQoL score in a specific group of older patients in the UK with probable sarcopenia – the group who form the target population for the use of this tool in clinical trials and other studies of sarcopenia. We found that the SarQoL tool had good internal consistency, with better consistency at the baseline visit (where SarQoL was administered face-to-face) than at the follow up assessment (where it was administered by telephone in almost all participants). Responsiveness to change was variable, with small numbers limiting the robustness of the analyses, but the SarQol questionnaire appeared to be more sensitive to improvement than to deterioration, with sample sizes of 25–100 required to detect clinically significant improvements from interventions. Only a weak correlation was seen between baseline and follow up SarQoL scores, suggesting that adjustments for baseline values of the SarQoL in analyses of clinical trial data are unlikely to improve trial power by a large amount. The moderate correlations between SarQoL and measures of physical performance give good evidence of concurrent validity; higher correlations would not be expected given that the constructs of physical performance and daily activities are related to, but distinct from, the construct of sarcopenia-related quality of life.

Our findings complement and extend previous development work on the SarQoL tool. Most previous validation studies for the SarQoL questionnaire have been conducted by the research group that originally designed the questionnaire, and the current analysis is one of the few independent validation studies that have been performed to date. A previous analysis in a UK-based population of healthy older people [7] (not selected for activity limitation or sarcopenia) showed good internal consistency (Cronbach’s alpha 0.88); SarQoL scores were lower in the small number of participants with sarcopenia than those without, and SarQoL scores showed close correlation with related domains (e.g. physical function) of other health status tools including the Short Form 36 (SF-36) questionnaire. Reliability was high (intraclass correlation coefficient 0.95), in part due to the short gap of only 2 weeks between testing and retesting. No attempt was made to test responsiveness to change. Similar results were obtained in older Belgian outpatients using similar methods [8].

One previous study used longitudinal follow-up data to test responsiveness in a small group of patients (*n* = 43) with sarcopenia [9], but this analysis relied on correlating change in SarQol over time with change in related health status tools (SF-36 and EuroQoL 5-dimension tool), rather than by using currently recommended anchor-based methods. Although these data showed good correlation between change in SarQoL and other measures over the two-year follow up period, these data support construct validity of the SarQoL rather than responsiveness per se, and do not enable derivation of a minimum clinically important difference. Pooled data from nine studies [10] was used to derive the smallest detectable change (estimated at 7 points); the test-retest interval in this analysis was short (2 weeks in all studies), and a standard-error based method was used which is not optimal for deriving the minimum clinically important difference [11].

Our study has a number of strengths. Firstly, we studied a group of older people with levels of physical function representative of those seen in primary and secondary care services, almost all of whom had a diagnosis of probable sarcopenia [12]. This group is the group for which a sarcopenia quality of life tool would be deployed in both research and clinical practice. We used a 6 month follow up interval for both baseline to follow-up correlation and for responsiveness testing. This interval reflects the interval between visits that would be used in clinical trials or between assessments in clinical practice. Such an interval also increases the chances that improvement or deterioration will have occurred. We used an anchor-based approach to determine responsiveness to change and to estimate minimum clinically important differences, reflecting current recommendations in this field.

A number of limitations of our analysis also require highlighting. The number of participants who improved between baseline and follow up was small. This likely reflects the natural history of sarcopenia but was also likely to be due to the effects of movement restrictions imposed to combat the COVID-19 pandemic [23]. We were unable to administer the SarQoL face-to-face for most participants at follow up because of the pandemic, and the lack of face-to-face visits also precluded collection of some of the other planned outcome measures, particularly physical performance measures. It is possible that the different mode of questionnaire delivery at follow-up may have introduced more variability in the scores, reducing the observed consistency or responsiveness. Conversely, consistency and responsiveness were still acceptable despite heterogeneity in the mode of administration, suggesting that in research or clinical practice participants could be given a choice of completing the questionnaire face-to-face or by telephone. We relied on natural change in sarcopenia over the six-month follow-up rather than the response to an intervention, and future studies should assess the responsiveness to change for the SarQoL after resistance training (the intervention with the best evidence for improving sarcopenia) [24, 25]. We elected not to measure reliability in this analysis as we did not re-administer the SarQoL within a sufficiently short period after the baseline visit to be confident that participants had remained clinically stable [26].

The responsiveness to change for the SarQoL may enable smaller sample sizes to be used in trials than some generic quality of life tools (the EQ-5D for instance typically requires sample sizes of 200–300 to enable detection of the minimum clinically important difference of 0.074 points [27]). However, disease-specific tools such as SarQoL complement, but cannot replace, generic health status or quality of life for older people. Such generic measures are still essential to assess broader health status in older people who will typically suffer from multiple long-term conditions [28].

Although previous studies have noted that the SarQoL took participants only 10 min to complete, our anecdotal experience in this study suggests that participants who are more functionally impaired may take longer. As only one of a battery of tests that might be administered during a trial or other study visit, it is worth considering if it is possible to reduce the burden on study participants by reducing the size of the questionnaire. Of note, the subdomain of ‘function’ within SarQoL delivers a similar distribution of normalised scores to the full SarQoL questionnaire and correlates highly with the total score; use of this subset of questions could potentially enable less burdensome data capture, albeit with the loss of some aspects of quality of life captured elsewhere in the full SarQoL. These findings parallel those from studies of other health status measures such as the SF-36, where the physical function subdomain shows a close correlation with grip strength [29]. Our results suggest that the SarQoL can be administered face-to-face and by telephone as well as by self-completion as studied previously; this flexibility of delivery is important for effective trial delivery both during a pandemic when face-to-face visits may be impossible, but also in non-pandemic times, when remote trial delivery can improve participation and retention [30].

## Conclusions

Taken together with previous validation studies of the SarQoL questionnaire, our results suggest that SarQoL has acceptable properties for use in clinical trials of sarcopenia interventions as part of a suite of outcomes. To date, few trials have included SarQoL as a trial outcome, and it is only by using SarQoL in clinical trials that we will be able to fully assess its performance as an outcome measure in this context.

## Data Availability

The datasets used and/or analysed during the current study are available from the corresponding author on reasonable request, subject to completion of a Data Access agreement with Newcastle University.

## Abbreviations

BIA: Bioimpedance analysis
EWGSOP: European Working Group on Sarcopenia in Older People
MCID: Minimum Clinically Important Difference
SARC-F: Strength, Assistance, Rise, Climb – Falls questionnaire
SarcNet: Sarcopenia Network and registry
SarQoL: Sarcopenia quality of life questionnaire
SD: Standard deviation
SF-36: Short-Form 36 questionnaire
SPPB: Short Physical Performance Battery
